# Quinoxaline 1,4-di-*N*-Oxides: Biological Activities and Mechanisms of Actions

**DOI:** 10.3389/fphar.2016.00064

**Published:** 2016-03-21

**Authors:** Guyue Cheng, Wei Sa, Chen Cao, Liangliang Guo, Haihong Hao, Zhenli Liu, Xu Wang, Zonghui Yuan

**Affiliations:** ^1^MOA Laboratory for Risk Assessment of Quality and Safety of Livestock and Poultry Products, Huazhong Agricultural UniversityWuhan, China; ^2^College of Veterinary Medicine, Huazhong Agricultural UniversityWuhan, China; ^3^National Reference Laboratory of Veterinary Drug Residues and MOA Key Laboratory for the Detection of Veterinary Drug Residues in Foods, Huazhong Agricultural UniversityWuhan, China

**Keywords:** quinoxaline 1, 4-di-*N*-oxides, antimicrobial, antitumoral, antiprotozoal, antiinflammatory, antioxidant, structure-activity relationship, mode of action

## Abstract

Quinoxaline 1,4-di-*N*-oxides (QdNOs) have manifold biological properties, including antimicrobial, antitumoral, antitrypanosomal and antiinflammatory/antioxidant activities. These diverse activities endow them broad applications and prospects in human and veterinary medicines. As QdNOs arouse widespread interest, the evaluation of their medicinal chemistry is still in progress. In the meantime, adverse effects have been reported in some of the QdNO derivatives. For example, genotoxicity and bacterial resistance have been found in QdNO antibacterial growth promoters, conferring urgent need for discovery of new QdNO drugs. However, the modes of actions of QdNOs are not fully understood, hindering the development and innovation of these promising compounds. Here, QdNOs are categorized based on the activities and usages, among which the antimicrobial activities are consist of antibacterial, antimycobacterial and anticandida activities, and the antiprotozoal activities include antitrypanosomal, antimalarial, antitrichomonas, and antiamoebic activities. The structure-activity relationship and the mode of actions of each type of activity of QdNOs are summarized, and the toxicity and the underlying mechanisms are also discussed, providing insight for the future research and development of these fascinating compounds.

## Introduction

Quinoxaline is a heterocyclic compound containing a benzene ring and a pyrazine ring. Oxidation of both nitrogens of the pyrazine ring to obtain QdNO offers it variety of biological properties, including antitumoral, antibacterial, anticandida, antitrypanosomal, antiinflammatory/antioxidant, and mutagenic properties. The versatile activities of QdNOs have aroused worldwide interests and endowed them potential application in human and veterinary medicines. For example, the QdNO antibacterial growth promoters have been widely used as feed additives to prevent infectious disease and improve animal growth since 1970s (Carta et al., 2005), and the antitumoral drug, TPZ (3-amino-BTO 1,4 dioxide, SR4233), has been subjected to phase II clinical trial (Covens et al., 2006; Maluf et al., 2006; Cohen et al., 2007). Although QdNOs with other activities are still in the research stage, they have exhibited great application prospects.

With the wide use of QdNOs, the toxicity and drug resistance gradually have become the disadvantages for further application of these promising compounds. The molecular targets of quinoxaline derivatives, which is a step beyond simply looking at their activities, should be analyzed. Nevertheless, knowledge about the mode of actions of QdNOs is far from clear, hindering the development of this kind of drugs. Therefore, a detail study on the mode of actions of QdNOs will provide information about the drug targets and the drug action pathway(s) and be helpful to construct models to screen new drugs. Meanwhile, the study of SAR combined with the study of drug action will explain the reason for that different structures of QdNOs exhibit different activities. Furthermore, since most of the drugs have more than one mechanism of action, the deeper study of the drug actions may discover new drug target(s) and provide potential approaches to conquer the problem of drug resistance and to mitigate or avoid toxicity. In this review, the mode of actions of QdNOs and the SAR analysis are elucidated and updated in sort of the different biological properties, giving insight to the future development of these fascinating compounds.

## Antimicrobial Activities of QdNOs

### Antibacterial Activity and Bacterial Resistance

**FIGURE 1:**
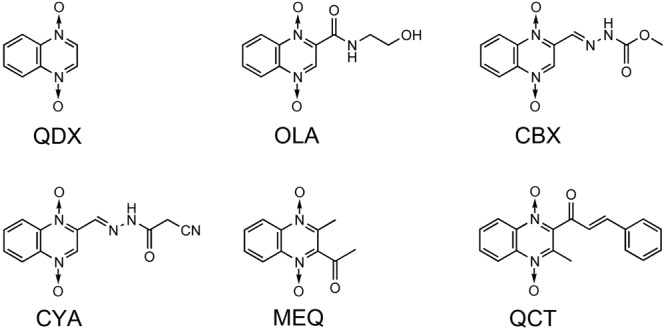
**Chemical structures of veterinary-used QdNO antibacterials**.

The antibacterial activity of QdNOs was first reported in McIlwain (1943), and subtherapeutic levels of antibacterial QdNOs have been used for nearly 50 years to promote the growth of animals and improve efficiency of feed conversion in animal husbandry (Carta et al., 2005). As shown in **Figure 1**, veterinary-used QdNO derivatives include (QdNOs), OLA [2-(*N*-2′-hydroxyethyl-carbamoyl)-3-methyl quinoxaline 1,4-di-*N*-oxide firstly synthesized by Bayer in 1967], CBX [hydrazine carboxylic acid (2-quinoxalinyl-methylene) methyl ester 1,4-di-*N*-oxide firstly synthesized by Pfizer in 1968]. MEQ (3-methyl-2- quinoxalinacetyl-1,4-di-*N*-oxide) and QCT (3-methyl-2-quinoxalinbenzenevinylketo-1,4-di-*N*-oxide) are novel synthetic QdNO derivatives developed by Lanzhou Institute of Animal Husbandry and Veterinary Drugs, Chinese Academy of Agricultural Sciences (Lanzhou, China). CYA [cyan acetic acid (1,4-di-*N*-oxy-quinoxalin-2-ylmethylene] hydrazide firstly designed by Chemapol Benelux) is a new QdNO member which is being evaluated in clinical trials for animals in China.

Olaquindox exhibits wide spectrum of antibacterial effect, strongly inhibiting the growth of *Escherichia coli, Proteus, Pasteurella*, and dysentery spirochete (Drumev, 1981). Because of its genotoxic potential, OLA is not permitted to use in food-producing animals in the European Union and Canada (WHO, 1994; European Union [EU], 1998; Standing Committee for Animal Nutrition [SCAN], 1998). CBX shows good therapeutic effect on swine dysentery caused by *Brachyspira hyodysenteriae* (Rainier et al., 1973a,b; Downing, 1974) and also has good antibacterial effects against *Salmonella, E. coli*, and other Gram-negative bacteria (Troutt et al., 1974; Das, 1984). CBX was once used as growth-promoting feed additives for piglets or pigs at growing phase (Yen and Pond, 1993) before it was prohibited by the European Union [EU] (1998) because of its mutagenic effects, developmental and reproductive toxicity and carcinogenicity (WHO, 1991; European Union [EU], 1998). In view of the significant effect of CBX on swine dysentery and bacterial enteritis, the United States, Canada, and other countries still allow it used as therapeutic agents. MEQ, which shows good antibacterial activity against Gram-negative bacteria, especially *Salmonella*, has been widely used in China as an animal feed additive and veterinary medication for diseases, such as swine dysentery and piglet white diarrhea (Liu et al., 2012). QCT is active against *B. hyodysenteriae*, and is also effective against *Salmonella, E. coli*, and other Gram-negative bacilli. In China, QCT is used as a growth promoting agent for pigs, poultry, and aquatic and has been approved as an animal growth promoter in China since 2003 (Ministry of Agriculture of P. R. China, 2003). CYA shows good antibacterial activity against *Staphylococcus hyicus, Pasteurella multocida*, and *E. coli* (Ding et al., 2006a,b), and also exhibits good growth-promoting effect on broilers and swine. With good clinical safety, CYA has been regarded as a potential replacement of OLA and CBX (Cihak and Srb, 1983).

Only a few studies have investigated the mechanism of antibacterial action of QdNOs. Suter et al. (1978) first found the synthesis of DNA (but not RNA and protein) was completely inhibited by QDX in the absence of oxygen. QdNOs also induced degradation of DNA in both proliferating and non-proliferating cells, while strains were more resistant in the presence of oxygen. QDX was reduced to quinoxaline-*N*-oxide by the intact *E. coli* cells or the cell-free extract. EPR measurements demonstrated the generation of free radicals during the reduction of QDX. Oxygen or deficiency of energy sources impaired the antibacterial activity and the reduction of QDX. In consistence with Suter’s result, our group recently found that CYA also had anaerobe-selective activity, and losing one or two of the oxygen’s of CYA exhibited no antibacterial activity (Cheng et al., 2015).

**FIGURE 2:**
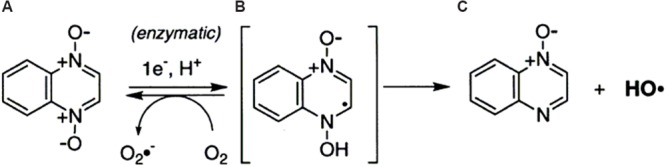
**One-electron reduction of quinoxaline 1,4-dioxide by reductase (Ganley et al., 2001)**.

The ability of QDX to cleave DNA was explicitly characterized using *in vitro* assays by Ganley et al. (2001). The results evidenced that QDX was a hypoxia-selective, redox-activated DNA-cleaving agent. The action of QDX on DNA yielded direct strand breaks with almost no sequence specificity, consistent with the involvement of radical species. In the absence of oxygen, QDX (**a**) received an electron and a hydrogen ion to form a neutral radical (**b**) (**Figure 2**). They considered two possibilities about the DNA-cleaving radical resulting from redox activation of QDX (**a**). First, the radical **b** might directly abstract hydrogen atoms from the DNA backbone, followed by elimination of water to yield the quinoxaline monoxide (**c**). Alternatively, radical **b** might fragment to form the known DNA-cleaving agent ^∙^OH and monoxide **c**.

Early study showed that *polA, recA, recB, recC, exrA*, and *uvrA E. coli* mutants were more susceptible to QdNOs than the corresponding repair-proficient strains, while the QdNO reductase activity was demonstrated to be lower in QdNO-resistant mutants than in the susceptible parent strain (Suter et al., 1978), indicating that QdNOs were reduced and caused extensive DNA damage in bacterial. Recently, our group investigated the transcriptomic and proteomic profiles of *E. coli* exposed to QdNOs and found that QdNOs mainly induced SOS response and oxidative stress (Cheng et al., 2015). We also confirmed that ROS were induced in the QdNO-treated bacteria and the free radical scavengers attenuated the antibacterial action of QdNOs and DNA damage, suggesting an oxidative-DNA-damage action of QdNOs. The QdNO radical intermediates, likely carbon-centered and aryl-type radicals, as identified by EPR, were the major radicals induced by QdNOs, and xanthine oxidase was identified as one of the QdNO-activating enzymes by using specific enzyme inhibitors (Cheng et al., 2015).

Tirapazamine is a prodrug undergoing clinical trials for various types of cancers (discussed in details in section “Antitumor activity of QdNOs”). Shah et al. (2013) showed that TPZ has antibacterial activity against *E. coli, S. aureus*, and *Clostridium difficile*, particularly at low oxygen levels. *E. coli* mutants deficient in HR were hypersusceptible to TPZ, suggesting that drug toxicity may be due to DNA damage. Moreover, *E. coli* strains deleted for genes encoding putative reductases were resistant to TPZ, implying that these enzymes are responsible for conversion of the prodrug to a toxic compound.

Though the two *N*-oxide groups are necessary for the antibacterial activity of QdNOs, some reduced form of quinoxaline compounds were still reported with antibacterial activity (Refaat et al., 2004; Singh et al., 2010, 2011). For example, when the C_2_ chlorine of 2-Chloro-3-methylquinoxaline was replaced with a benzene ring harboring an aldehyde or a free amino group which can be further reacted with aromatic amines and aromatic aldehydes, this compounds also show antimicrobial activity (Singh et al., 2010). Therefore, in addition to the *N*-oxide group, the side chain of quinoxaline is another determinant of activity.

Hansen et al. (2004) first reported the gene-encoded resistance mechanism to OLA. In their study, two genes of *oqxA* and *oqxB*, encoding for proteins homologous to resistance-nodulation-cell-division family eﬄux systems, were cloned from a conjugative plasmid isolated from *E. coli*. Plasmids containing the *oqxAB* genes yielded high resistance to OLA and chloramphenicol in *E. coli*, indicating *oqxAB* encodes a multidrug eﬄux pump. Later, they demonstrated the prevalence of the OqxAB eﬄux pump by horizontal transfer of OLA resistance from OLA-resistant isolates using an OLA-sensitive *E. coli* as recipient (Hansen et al., 2005). In addition to OLA and chloramphenicol, the OqxAB pump conferred antimicrobial resistance or reduced susceptibility toward a variety of substrates in *E. coli*, including animal growth promoters, antimicrobials, disinfectants and detergents (Hansen et al., 2007). Interestingly, *oqxA* gene was not detected in the CYA/OLA- resistant *E. coli* induced *in vitro* (Guo et al., 2012), suggesting there are other mechanisms conferring the bacterial resistance.

### Antimycobacterial Activity

Tuberculosis is a common, and in many cases lethal, infectious disease caused by various strains of mycobacteria, usually *Mycobacterium tuberculosis*. TB has been a companion of mankind since the beginning of human history. In 2010, there where 8.8 million new cases, and 1.5 million deaths, mostly in developing countries (WHO, 2011), where more people contract TB because of compromised immunity due to high rates of AIDS (Lawn and Zumla, 2011). Drug-resistant TB is a public health issue in many developing countries, as treatment of it is longer and requires more expensive drugs. MDR TB is defined as resistance to the two most effective first-line TB drugs, rifampicin and isoniazid. Over the past years, QdNO derivatives as antitubercular drug candidates hold promise in the treatment of TB and its resistant form.

**FIGURE 3:**
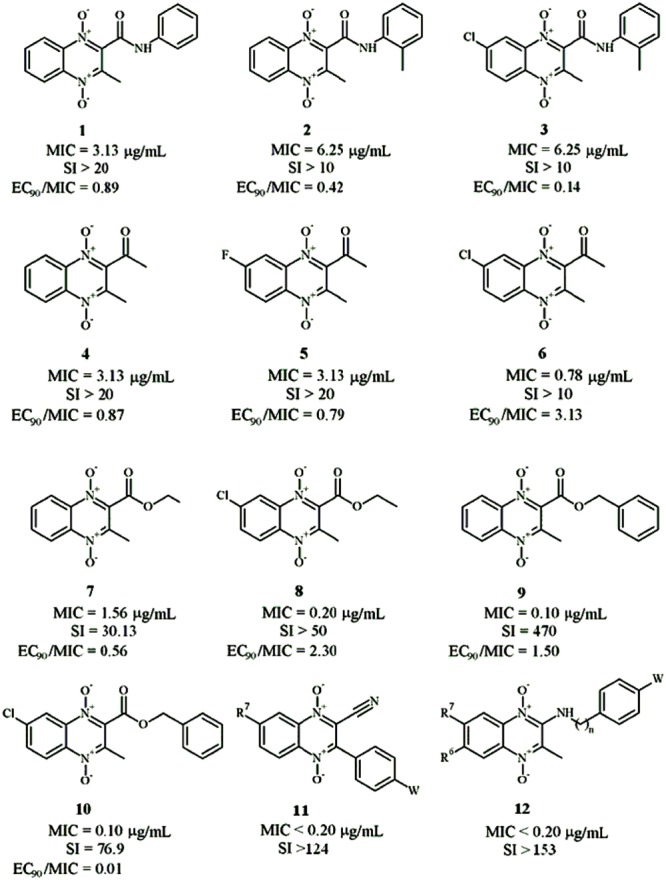
**General structures of QdNO derivatives with antimycobacterial activity published by Monge’s team [modified from Vicente et al. (2011)]**.

The first publication of QdNO derivatives as antimycobacterium agents dates back to the end of the 1990s by Monge’s team (Montoya et al., 1998). Continued identification of novel antitubercular candidates based on their potency (MIC to H_37_Rv *M. tb*), selectivity [SI = IC_50_(to VERO cell)/MIC(to H_37_Rv *M. tb*)] and low cytotoxicity make them valid new leads for synthesizing additional analogs with improved antitubercular activity both *in vitro* and *in vivo*. A comprehensive review on the properties of QdNO derivatives developed as potential antitubercular agents by Monge’s team was reported recently (Vicente et al., 2011). As shown in **Figure 3**, the QdNO antitubercular compounds can be divided into categories based on their structures, including amide derivatives (**1–3**), ketone derivatives (**4–6**), ester derivatives (**7–10**) and other derivatives (**11–12**). Compounds **8** and **10**, proved efficacious *in vivo* in a murine model of low dose aerosol infection. Moreover, these two compounds also showed activity against non-replicating bacteria, indicating that QdNOs might lead to shortened therapy, because non-replicating bacteria is believed to be a major factor responsible for the prolonged nature of antitubercular therapy. Compound **8** is also active on single-drug resistant and MDR clinical isolates.

**FIGURE 4:**
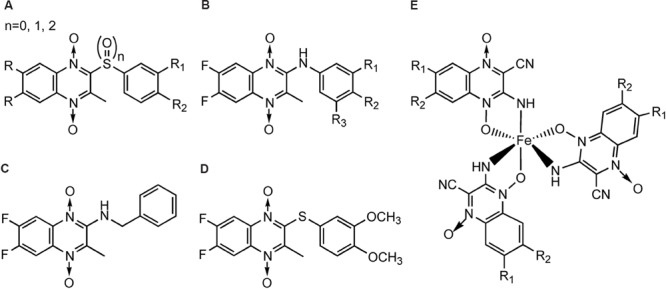
**General structures of QdNOs with antimycobacterial activity published by Zanetti’s **(A–D)** and Gambino’s teams (E)**.

In the meantime, there were other research groups studying the antimycobacterial activity of QdNOs. As shown in **Figure 4**, Zanetti’s team reported a series of 3-methylquinoxaline 1,4-di-*N*-oxide derivatives with a phenylthio, phenylsulfonyl, or phenylsulfinyl linked in R2 position of quinoxaline subunit (**Figure 4A**). The series of 3-methyl-2-phenyl-thioquinoxaline 1,4-di-*N*-oxide derivatives presented the best MIC data, ranging between 0.39 and 0.78 g/mL, whereas the oxidation of sulfur bridge to yield phenylsulfinyl and phenylsulfonyl derivatives or its replacement with benzylamino (**Figure 4B**) or phenylamino group (**Figure 4C**) slightly reduces its activity (Carta et al., 2002, 2004). Furthermore, 1 mg/L of 6,7-difluoro-3-methyl-2-(3,4-dimethoxyphenylthio) quinoxaline 1,4-dioxide (**Figure 4D**) exhibited good activity in mouse macrophages infected by *M. avium paratuberculosis* (Zanetti et al., 2005).

Gambino’s team has devoted to the synthesis and biological assessment of a large amount of QdNO derivatives complexed with metals, including palladium (Pd; Urquiola et al., 2009) and copper (Cu; Torre et al., 2005; Urquiola et al., 2008) with antitumoral activity, vanadium (Vo) with antitrypanosomal (Urquiola et al., 2006), insulin-mimetic (Noblia et al., 2006), and antitumoral activities (Vieites et al., 2006), and iron (Fe) with antimycobacterial activity (Tarallo et al., 2008, 2010). Two novel iron complexes, [Fe(L3)(3)] (L3: R1 = Cl, R2 = OCH3) and [Fe(L4)(3)] (L4: R1 = H, R2 = CF3; **Figure 4E**) showed *in vitro* growth inhibitory activity on *M. tuberculosis* H(37)Rv (MIC = 0.78 μg/mL), together with very low unspecific cytotoxicity on murine cell line J774. Both complexes showed higher inhibitory effects on *M. tuberculosis* than the second-line therapeutic drugs (Tarallo et al., 2010).

Structure-activity relationship suggests that the 1,4-di-*N*-oxide groups in quinoxaline ring are important to increase the antimycobacterial activity (Montoya et al., 1998; Ortega et al., 1999; Sainz et al., 1999). Quinoxaline-2-carbonitrile derivatives has good antimycobacterial activity but appeared to be quite toxic (Ortega et al., 2001, 2002); thus, the replacement of the carbonitrile group with a carboxamide (Zarranz et al., 2003), acetyl, benzoyl (Jaso et al., 2003), or carboxylate groups (Jaso et al., 2005) was proposed. Among the 6(7)-substituted quinoxaline-2-carboxylate 1,4-dioxide derivatives, anti-TB activity principally depends on the substituents in the carboxylate group, improving in the following order: benzyl > ethyl > 2-methoxyethyl > allyl > tert-butyl, and the presence of a chloro, methyl, or methoxy group in position 7 of the benzene moiety reduces the MIC and IC(50) values (Jaso et al., 2005). Recently, a research employed 3D-QSAR and docking analysis to identify molecular structural features required for effective antimycobacterial activity of quinoxaline-2-carboxamide 1,4-di-*N*-oxide derivatives inside the active site of the *Mycobacterium* DNA gyrase B subunit, and the obtained binding mode was as same as that of the novobiocin X-ray structure (Radwan and Abdel-Mageed, 2014).

### Antifungal Activity

*Candida* is a genus of yeasts, some of which can cause disease, for example, *Candida albicans* cause infections (candidiasis or thrush) in humans and other animals. In healthy individuals, these infections can be cured with topical or systemic antifungal medications (commonly over-the-counter treatments like miconazole or clotrimazole), while in debilitated or immunocompromised patients, candidiasis may become a systemic disease.

**FIGURE 5:**
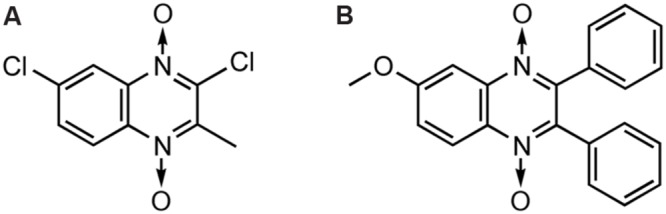
**General structures of QdNOs with antifungal activity**.

Carta et al. (2002, 2004) reported the *in vitro* anticandida activity (against *C. albicans, C. glabrata, C. krusei*, and *C. parapsilosis* clinical isolates) of 3-methylquinoxaline 1,4-dioxide derivatives, in particular 2,7-dichloro-3-methyl quinoxaline 1,4-dioxide (**Figure 5A**) and 2-chloro-7-ethoxy-6-fluoro-3-methylquinoxaline 1,4-dioxide were the most active against *C. Krusei*, exhibiting MICs of 0.4 and 1.9 g/mL, respectively (miconazole MIC = 0.9 g/mL). Murthy et alreported one of the 2,3-diphenyl quinoxaline 1,4-dioxide derivatives (**Figure 5B**) as a fruitful matrix for further biological evaluation based on its wide zones of inhibition against *C. albicans* and *S. cerevisiae* (Murthy et al., 2011).

## Antitumor Activity Of QdNOs

It has been recognized for more than 50 years that very low levels of oxygenation (hypoxia) protect cells from killing by X-irradiation. Hypoxic cells, which are resistant to radiotherapy, are present in solid tumors but not in normal tissues, therefore become a major problem for radiation therapy of tumors. Additionally, hypoxic cells are non-proliferating which do not respond to drugs active against only proliferating cells, and are distant from the blood vessels carrying the drug. Thus the development of drugs with selective toxicity toward hypoxic cells is a key objective in anticancer chemotherapy. The recent development of QdNOs that are non-toxic until they are activated in the hypoxic cell opens a new era.

**FIGURE 6:**
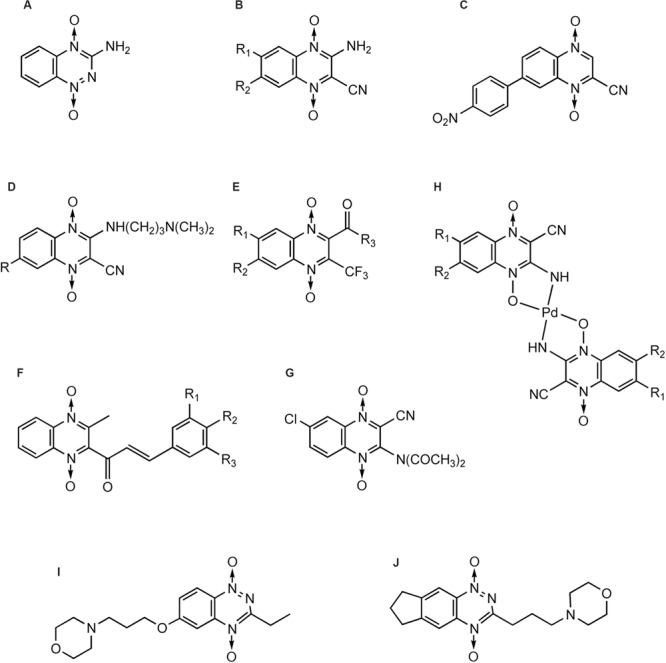
**QdNOs with hypoxia-selective and antitumoral activities**.

Tirapazamine (Zeman et al., 1986) (**Figure 6A**), which actually belongs to BTOs, is the first drug that has been shown to be an efficient and selective cytotoxin for hypoxic cells. TPZ has been subjected to phase II testing in patients with head, neck, and gynecological cancers (Covens et al., 2006; Maluf et al., 2006; Cohen et al., 2007).

In addition to TPZ, many research teams have been devoted to the synthesis and development of QdNO antitumoral drugs. Monge et al. (1995b) synthesized a series of 3-aminoquinoxaline-2-carbonitrile 1,4-di-*N*-oxides (**Figure 6B**) with a range of electron-donating and -withdrawing substituents in the 6- and/or 7- positions and evaluated for toxicity to hypoxic cells. As the electron-withdrawing nature of the 6(7)-substituent increases, the reduction potential becomes more positive and the compound is more readily reduced. The most potent cytotoxins were the 6,7-dichloro and 6,7-difluoro derivatives, which were 30-fold more potent than TPZ. The compound 7-(4-nitrophenyl)-2-quinoxalinecarbonitrile 1,4-di-*N*-oxide (**Figure 6C**) is 150-fold more potent than TPZ, demonstrating that the amino group in three position is not necessary for activity (Monge et al., 1995a). Meanwhile, they developed two derivatives bearing a basic side chain. The 7-choloro- and 7-trifluoromethyl 3-(dimethylaminopropyl) amino-2-quinoxalinecarbonitrile 1,4-dioxide (**Figure 6D**) showed high potency and excellent selectivity (Monge et al., 1995a). They further studied the activity of the basic chain in 3 position of the 2-quinoxalinecarbonitrile 1,4-dioxides and showed that the replacement of (*N,N*-dialkyl amino) alkyl amino chain with aromatic rigid moieties (anilines and arylpiperazines) in three position held the potency but reduced the hypoxia-selectivity (Ortega et al., 2000). Continuingly, they synthesized a new series of 2-alkylcarbonyl and 2-benzoyl-3-trifluoromethylquinoxaline 1,4-di-*N*-oxide (**Figure 6E**) and evaluated for *in vitro* antitumor activity against MCF7 (breast), NCI-H460 (lung), and SF-268 (central nervous system) cells. In general, anticancer activity depends on the substituents in the carbonyl group, improving in the order: ethyl < isopropyl < tert-butyl < phenyl-ones (Zarranz et al., 2004). By comparison of the ^1^H NMR spectra, Solano et al. (2007) also showed that the best activity was observed in derivatives with electron-withdrawing groups in position 6 or 7 on the quinoxaline ring whereas loss of one or two oxygens reduced the cytotoxicity.

Besides Monge’s team, Das et al. (2009) reported a series of 2-(3-aryl-2-propenoyl)-3-methylquinoxaline-1,4-dioxides (**Figure 6F**) could reverse the MDR properties of murine L-5178Y leukemic cells which were transfected with the human MDR1 gene. Ismail et al. (2010) evaluated antitumor activity of a new series of QdNOs against liver carcinoma (Hepg2) and brain tumor (U251) human cell line, and compound 4 (**Figure 6G**) was the most potent hypoxia selective-cytotoxin on EAC cell line (Ismail et al., 2010). Urquiola et al. (2009) synthesized four new palladium(II) complexes with the formula Pd(L)(2) (**Figure 6H**), where L were quinoxaline-2-carbonitrile 1,4-di-*N*-oxide derivatives, and evaluated their cytotoxicity on V79 cells. Pd(L1)(2) and Pd(L2)(2), where L1 was 3-aminoquinoxaline-2-carbonitrile 1,4-di-*N*-oxide and L2 was 3-amino-6(7)-methylquinoxaline-2-carbonitrile 1,4-di-*N*-oxide, showed non-selective cytotoxicity. Pd(L3)(2), where L3 was 3-amino-6(7)-chloroquinoxaline-2-carbonitrile 1,4-di-*N*-oxide, resulted *in vitro* more potent cytotoxin in hypoxia than the corresponding free ligand and TPZ. Pd(L2)(2) introduced a scission event in supercoiled DNA, yielding the circular relaxed form. Meanwhile, both Pd (L1)(2) and Pd(L3)(2) produced the loss of negative supercoils, rendering a family of topoisomers with reduced electrophoretic mobility. For the highest doses assayed, Pd(L3)(2) was even able to introduce positive supercoils on the plasmid DNA (Urquiola et al., 2009).

Although TPZ has attractive features of targeting hypoxic cells in tumors, it has limited clinical activity, in part because of poor extravascular penetration. Hicks et al. (2010) used a spatially resolved pharmacokinetic/pharmacodynamic model to guide the progression of 281 TPZ analogs through a hierarchical screen. SN29751 (**Figure 6I**) and SN30000 (**Figure 6J**) were identified as the most promising hypoxic cytotoxins, and SN30000, in particular, showed higher hypoxic potency and selectivity than TPZ in tumor cell cultures and faster diffusion through HT29 and SiHa multicellular layers.

### QdNO-Induced Radicals

**FIGURE 7:**
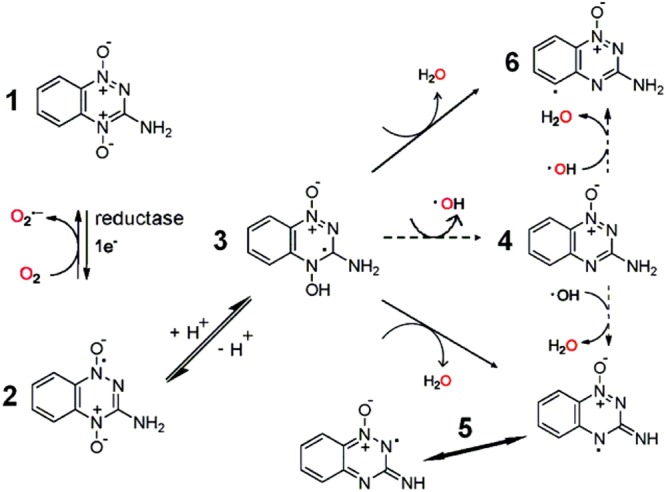
**Bioreduction of TPZ with produced free radicals based on Shinde et al. (2010) and Yin et al. (2012)**.

The mode of action of QdNO antitumoral drugs was most comprehensively studied in TPZ. As shown in **Figure 7**, it is known that TPZ (**1**) is an excellent substrate for a variety of intracellular reductases that add an electron to the drug to form an radical anion (**2**) (Brown, 1999), which then receives a proton to form a neutral radical (**3**). This neutral radical itself or produces other active radicals to abstract hydrogen from DNA, producing both SSBs and DSBs, resulting in chromosome breaks (Wang et al., 1992). Under aerobic conditions, oxygen can remove the additional electron from the radical anion (**2**), thereby back-oxidizing it to the non-toxic parent with a concomitant production of $O_{2}^{•–}$ (Lloyd et al., 1991). Thus, the differential hypoxic cytotoxicity results from the fact that the TPZ radical is much more cytotoxic than the $O_{2}^{•–}$.

There have been a number of suggestions about the identity of active radicals produced by TPZ. An early proposal was that the ^∙^OH radical was released from the protonated anion (**3**), and the monoxide of TPZ (**4**) was produced simultaneously (Laderoute and Rauth, 1986), but this was later rejected in favor of the protonated radical anion itself as being the active species (Laderoute et al., 1988). Release of an ^∙^OH radical has been favored by other research workers, based on the similarity in the spectrum of products arising from TPZ-mediated damage to both purine and pyrimidine bases and deoxyribose sugars when compared with damage arising from the ^∙^OH radical, although TPZ-mediated damage exhibited some preference for purine over pyrimidine damage (Kotandeniya et al., 2002; Birincioglu et al., 2003; Chowdhury et al., 2007). Additionally, an EPR study using DMPO as a spin trap reported a composite spectrum of both a C-centered and an OH-adduct, DMPO-OH (Patterson and Taiwo, 2000), supporting evidence for the release of an ^∙^OH radical.

In contrast, Shinde et al. (2009, 2010) could not find any evidence that ^∙^OH is eliminated from TPZ following enzymatic reduction. They presented that the protonated radical anion (**3**) undergoes dehydration to the BTZ (**5**) (Shinde et al., 2009), causing oxidative damage to DNA and to oxidize TPZ itself (Anderson et al., 2003a,b, 2006). *In situ* EPR measurement confirmed that a multi *N*-centered radical, consistent with the BTZ radical (**5**), was formed (Shinde et al., 2009). Similarly, the one electron reduction potentials [E(1)R] of the BTZ radicals (**5**) also tracked the hypoxic cytotoxicity to human tumor cells in a series of BTO analogs of TPZ (3-amino BTO; Anderson et al., 2005). However, although the E(1)R value for the BTZ radical (**5**) of TPZ, 1.31 V (Anderson et al., 2003b), is sufficient to oxidize the purine bases of DNA, but not to directly oxidize the pyrimidine bases of DNA. Later, they trapped TPZ and a series of 3-substituted analogs by *N*-tert-butyl-*R*-phenylnitrone and observed by EPR the formation of their aryl (phenyl) radicals (**6**), which is stronger oxidants than the BTZ radical (**5**) (Shinde et al., 2010).

There are still some questions whether protonated radical anion (**3**) will undergo N–OH homolysis to its monoxide (**4**) and ^∙^OH, or alternatively, radical anion (**2**) may react by dehydration and form aminyl radicals (**5**) and H_2_O or phenyl radicals (**6**) and H_2_O. Recently, Yin et al. (2012) discovered that dehydration might be the result of a two-step sequence that involved N–OH homolysis and formation of ^∙^OH aggregates of the monoxide (**4**) followed by H-abstraction within the ^∙^OH aggregates to form hydrates of aminyl (**5**) or of phenyl (**6**) radicals (**Figure 7**, dashed arrows).

Electron paramagnetic resonance experiments indicate both an aryl-type radical and an oxidizing radical, trapped as a carbon-centered radical, are formed from the protonated radical anion of the bioreductive anticancer prodrug, SN30000 (Anderson et al., 2014). The carbon-centered radical, produced upon the one-electron oxidation of the 2-electron reduced metabolite of SN30000, oxidizes 2-deoxyribose, leads to double strand breaks. Hunter et al. (2014a) found hypoxia-activated prodrugs of DNA-damaging cytotoxins (TPZ, SN30000) might inhibit growth of triple-negative breast cancer (TNBC) by simultaneously addressing the two targets, tumor hypoxia and derangement of HR repair.

Recently, Yadav et al. (2014) found The ^∙^OH radical was released from 3-trifluoromethyl-quinoxaline 1,4-dioxides upon one-electron reduction by cytochrome P450 oxidoreductase. This process effectively competes with back oxidation of the intermediate radical anion by oxygen and underlies the increased aerobic cytotoxicity of such compounds compared to that seen for, TPZ.

### Reductase

Another major unresolved question is the identity of the enzyme(s) that activates a QdNO to cause cell death. Numerous enzymes, including xanthine oxidase (EC 1.2.3.1; Laderoute et al., 1988), cytochrome P450 (Wang et al., 1993), DT-diaphorase (EC 1.6.99.2; Patterson et al., 1994), and NADPH:cytochrome P450 reductase (EC 1.6.2.4; Patterson et al., 1995), are able to metabolize TPZ *in vitro* under hypoxic conditions. A correlation between NADPH:cytochrome P450 reductase level (Patterson et al., 1997) or NOS (Chinje et al., 2003) level and sensitivity to TPZ has been reported. For a long time, there was no agreement as to which enzyme(s) are involved in the DNA damage until Evans et al. (1998) showed that TPZ was metabolized to DNA-damaging radicals by intra-nuclear enzymes. TPZ radicals formed outside nuclei do not contribute to intranuclear DNA damage, and the 80% of the drug metabolism that occurs in the cytoplasm is probably irrelevant for hypoxic killing effect of this drug. Later, Delahoussaye et al. (2001) demonstrated that multiple reductases in the nuclear matrix metabolized TPZ under hypoxia. DNA SSBs were probably caused by the most abundant source of reductase in the nucleus, while DNA DSBs were formed by an unknown nuclear reductase requiring only NADPH for its activity.

Hunter et al. (2014b) observed that forced expression of FAD-dependent oxidoreductase domain containing 2 (FOXRED2) increased activation of hypoxia-targeted prodrugs TPZ and SN30000. They also identified the flavoprotein P450 (cytochrome) oxidoreductase, which is responsible for prodrug activation during hypoxia, as the predominant determinant of sensitivity to SN30000 (Hunter et al., 2015).

### Topoisomerase and Polymerase β

Topoisomerase II is essential in mammalian cells, because it resolves the unfavorable topological structures in DNA during replication and transcription. Drugs that stabilize Topo II with a DNA DSB to form cleavable complex are termed Topo II poisons (Pommier et al., 2010). Since TPZ produces a marked inhibition of DNA replication in the nuclear matrix (Peters et al., 2001), and TPZ-induced DNA DSBs are protein-associated (Olive, 1995; Siim et al., 1996), Peters and Brown (2002) considered TPZ might poison Topo II. They found under hypoxic conditions, the nuclear extracts from LXFL 529 human lung carcinoma cells treated with TPZ reduced the activity of Topo II. Inhibitors of the Topo II catalytic cycle abrogated TPZ-generated DNA DSBs and cytotoxicity, and TPZ stabilized DNA Topo II cleavable complexes (Peters and Brown, 2002). Using *Saccharomyces cerevisiae* as a model, overexpression of TOP2 (encoding Topo II) leads to hypersensitivity to TPZ, suggesting that Topo II is also a target of TPZ in yeast (Hellauer et al., 2005). XK469 (NSC 697887) and CQS (NSC 339004), two synthetic quinoxaline derivatives without two oxygens on the quinoxaline ring, also show solid tumor selectivity. XK469 and CQS have entered Phase I and Phase II clinical study, respectively (Miller et al., 1997; Bekaii-Saab et al., 2006; Alousi et al., 2007). Gao et al. (1999) reported the primary target of XK469 is Topo IIβ, and CQS was found to be both a Topo-IIα and a Topo-IIβ poison (Gao et al., 2000). The large aromatic side chains of XK469 and CQS can not be ignored to the drug action. Except for TopII, TPZ also induces other DNA-protein cross-links, including DNA-Topo I cleavable complexes (Evans et al., 2008) and DNA-Polβ cross-link with the lesion (Sung and Demple, 2006).

Evans et al. (2008) further showed an overall model of TPZ damage in which DNA SSBs, base damage, and DNA-protein cross-links (including Topo I and II cleavable complexes) produced stalling and collapse of replication forks. The resolution of the complex required HR and XPF/ERCC1 protein for their repair. Cells defective in HR proteins were particularly sensitive to TPZ, and extensive sister chromatid exchanges occurred after treatment with TPZ. In addition, TPZ preferentially kills mutants both with defects in XPF/ERCC1 and base excision repair. H2AX, an indicator of DNA DSBs, is induced preferentially in cells in the S phase of the cell cycle.

In a dose-dependent fashion, polymerase β DNA-protein cross-links (Polβ-DPC) were detected in MDA-MB-231 human cells treated with the antitumor drug TPZ (much more Polβ-DPC under 1% O_2_ than under 21% O_2_; Quinones et al., 2015). Mouse embryonic fibroblasts challenged with TPZ also incurred Polβ-DPC.

### Hypoxia-Inducible Factor 1

Hypoxia-inducible factor 1, a heterodimeric transcription factor that mediates the adaptation of tumor cells and tissues to the hypoxic environment, has attracted considerable interest as a potential therapeutic target. The subunit 1α of HIF plays an essential role in the transcriptional activation of genes involved in tumor angiogenesis invasiveness and metastasis with its downstream target, VEGF (Carmeliet et al., 1998).

Quinoxaline 1,4-di-*N*-oxides could reduce the expression of HIF-1α mRNA in T-84 cells, and 2-benzoyl-3-phenyl-6,7-dichloroquinoxaline 1,4-dioxide (DCQ) is shown most effective in decreasing the HIF-1α mRNA and protein levels (Diab-Assef et al., 2002). A hypoxic cytotoxin, 3-amino-2-quinoxalinecarbonitrile 1,4-dioxide (TX-402), which is an improved analog of TPZ, has been shown to inhibit HIF-1α expression (Nagasawa et al., 2003). Recently, it is found that TPZ acts in a novel manner to inhibit HIF-1α accumulation, in which HIF-1α translational regulation is involving, dependent on the phosphorylation of translation initiation factor 2a (eIF2a; Zhang et al., 2010).

Weng et al. (2011) showed that suppression of HIF-1α by 3-(4-Bromophenyl)-2-(ethylsulfonyl)-6-methyl-quinoxaline 1,4-dioxide (Q39) resulted in a drastic decrease in VEGF expression. Unlike TPZ, suppression of HIF-1α accumulation by Q39 correlated with prominent dephosphorylation of mTOR (mammalian target of rapamycin) and initiation factor 4E-binding protein 1 at the translational level. 3-[2-hydroxyethyl(methyl)amino]-2-quinoxalinecarbonitrile 1,4-dioxide (TX-2098) was also shown to have antitumor effect in pancreatic cancer, through inhibiting VEGF and HIF-1α targeted gene expression (Miyake et al., 2012). Recently, it is demonstrated that DCQ blocks breast cancer metastasis by targeting the HIF-1 pathway (Ghattass et al., 2014). Cancer cell death was associated with an increase in ROS independently of p53 and was inhibited by antioxidants. DCQ-induced ROS was associated with DNA damage, the downregulation of HIF-1α, and inhibition of VEGF secretion. In MCF-7 (p53 wildtype), HIF-1α inhibition was partially via p53-activation and was accompanied by a decrease in p-mTOR protein, suggesting interference with HIF-1α translation. In MDA-MB-231 (p53 mutant), DCQ reduced HIF-1α through proteasomal-dependent degradation mechanisms.

Combinational use of TPZ with other inhibitors represents a novel mechanism for targeting tumor. In combination with TPZ, topoisomerase I inhibitors exhibited synergistic cytotoxicity and induced significant apoptosis in several hepatocellular carcinoma cell lines (Cai et al., 2014). The enhanced apoptosis induced by TPZ plus SN-38 (the active metabolite of irinotecan) was accompanied by increased mitochondrial depolarization and caspase pathway activation. The combination treatment dramatically inhibited the accumulation of HIF-1α protein, decreased the HIF-1α transcriptional activation, and impaired the phosphorylation of proteins involved in the HR repair pathway, ultimately resulting in the synergism of these two drugs. TPZ mediates central vascular dysfunction in tumors and is also a competitive inhibitor of NOS. Baker et al. (2013) further investigated the vascular-targeting activity of TPZ by combining it with NOS inhibitor L-NNA, or with low oxygen content gas breathing. Irreversible loss of perfusion and enhanced tumor cell death was observed when TPZ was combined with either low oxygen or a NOS inhibitor, illustrating a novel use of hypoxia-activated cytotoxic prodrugs as vascular targeting agents.

## Antiprotozoal Activities Of QdNOs

### Antitrypanosomal Activity

*Trypanosoma cruzi* is the haemoflagellate protozoan that causes the Human American trypanosomiasis, or Chagas disease, representing a relevant health problem in Central and South America. The acute form of Chagas disease usually goes unnoticed and may present as a localized swelling at the site of entry. The chronic form may develop 10 to 20 years after infection, which affects internal organs (e.g., the heart, the esophagus, the colon, and the peripheral nervous system), sometimes causing death to affected people from heart failure. The first line of treatment is nifurtimox and benznidazole.

**FIGURE 8:**
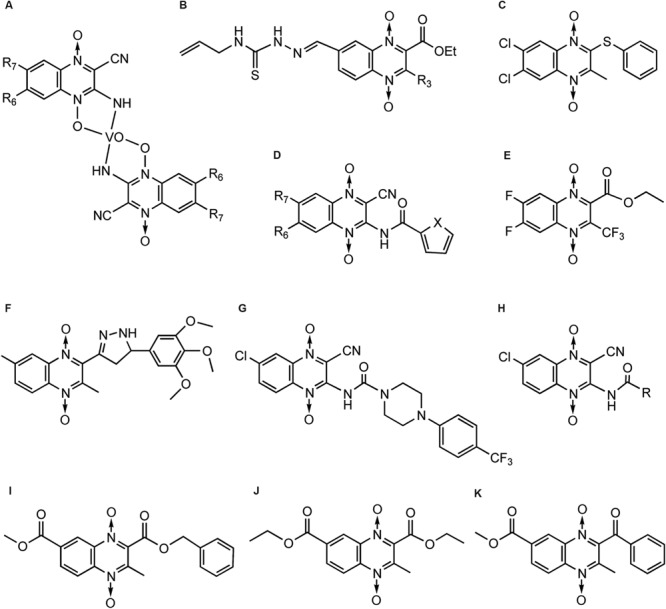
**QdNOs with antitrypanosomal activity**.

Cerecetto’s team (Cerecetto et al., 1999) firstly noticed QdNO derivatives had antitrypanosomal activity against epimastigote forms of *T. cruzi*. They reported a vanadium complexe of QdNO, [V(IV)O(L)(2)], where L were 3-aminoquinoxaline-2-carbonitrile 1,4-di-*N*-oxide derivatives (**Figure 8A**), was provide with excellent antitrypanosomal activity, similar to that of the reference drugs nifurtimox and benznidazole and higher than that of the corresponding free ligands. The antitrypanosomal activity of these vanadium complexes could be explained on the basis of their lipophilicity and the electronic characteristics of the quinoxaline substituents (Urquiola et al., 2006). They also reported two QdNO compound, R3 = methyl (SI > 53.3, selectivity index = ID_50_(macrophage)/ID_50_(*T. cruzi*)) and R3 = phenyl (SI > 33.3; **Figure 8B**), displayed excellent parasite/mammal selectivity indexes. Those compounds are able to accumulate squalene suggesting that anti-*T. cruzi* mechanism of action is not involved the inhibition of sterol biosynthesis (Gerpe et al., 2010).

Monge’s team also found that QdNO derivatives presented good inhibitor activity to the growth of *T. cruzi* in *in vitro* assays, among which a QdNO derivative (**Figure 8C**) were the most cytotoxic compounds against the protozoan, and the IC_50_ were of the same order of that of nifurtimox (Aguirre et al., 2004). The authors reported some SAR deductions: the presence of a halo-substituent (electron-withdrawing) at benzene moiety of quinoxaline produces more active compounds; more hydrophilic compounds decrease the antitrypanosomal activity; a reductive metabolism could be implicated in the mechanism of action. Later, they prepared a series of heterocyclic-2-carboxylic acid (3-cyano-1,4-di-*N*-oxidequinoxalin-2-yl)amide derivatives (**Figure 8D**), among which compounds (R6 = F, R7 = F, X = O) and (R6 = F, R7 = F, X = S; **Figure 8D**) presented IC_50_ values in the same order as nifurtimox (Ancizu et al., 2009). Recently, they synthesized a series of 3-trifluoromethyl QdNOs, among which derivatives possessing electron-withdrawing substituents in the 2-, 3-, 6-, and 7-positions were the most active compounds (Benitez et al., 2011). One 3-trifluoromethyl QdNO compound (**Figure 8E**), which was substituted with fluoro groups at the 6- and 7-positions of the quinoxaline ring, was the most active (IC_50_= 0.4 μM) and selective (*SI* = 10) in the cytotoxicity assay (Torres et al., 2013). It is demonstrated that inhibition of mitochondrial dehydrogenases are involved in the anti-*T. cruzi* activity of the most active derivatives (Benitez et al., 2011; Torres et al., 2013).

Leishmaniasis is a parasitic disease which appears in visceral, cutaneous and mucocutaneous forms affecting millions of people throughout the world. Monge’s team tested pyrazole quinoxaline derivatives against *Leishmania peruviana*. 2,6-dimethyl-3-f-quinoxaline 1,4-dioxide (**Figure 8F**) was the most active compound of this series against *L. peruviana* (IC_50_= 8.9 μM; Estevez et al., 2011). This compound resulted non-toxic for Vero and LLc-Mk2 cells, and was almost 6 to 13 times more active on *Leishmania* than on THP-1 or MPM. According to the study of Barea et al. (2012), piperazine linked QdNO (**Figure 8G**) emerged as the best leishmanicidal agent against *L. infantum* (IC_50_= 5.7 μM). Their later study indicated that the R = cyclohexyl derivative (**Figure 8H**) had the best antileishmanial activity against *L. infantum* (IC_50_= 2.5 μM) while the R = 3-chloropropyl derivative (**Figure 8H**) was the best against *L. amazonensis* (IC_50_= 0.7 μM; Barea et al., 2013). Villalobos-Rocha et al. (2014) evaluated the *in vitro* biological activity of 33 ethyl and methyl quinoxaline-7-carboxylate 1,4-di-*N*-oxide derivatives on *T. cruzi* and *L. mexicana*, of which M7 (**Figure 8I**) and E4 (**Figure 8J**) displayed activity against both parasites. Compound M2 (**Figure 8K**) was predicted in the docking procedure as a potential *T. cruzi* trypanothione reductase inhibitor by its interaction with five residues close to the active site of the enzyme.

### Antimalarial Activity

*Plasmodium falciparum* is one of the species of *Plasmodium* that cause malaria in humans. Malaria caused by this species is the most dangerous form of malaria, with the highest rates of complications and mortality. For nearly half a century, chloroquine has been the primary therapy of choice. However, chloroquine-resistant *P. falciparum* is now observed in nearly all of the malaria-endemic regions and causes the most deadly form of malaria. Therefore, it is necessary to develop cheaper and more effective drugs against the parasite.

**FIGURE 9:**
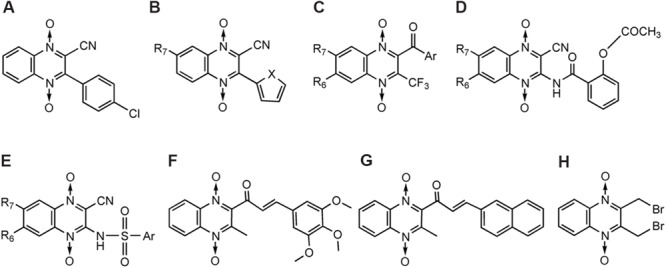
**QdNOs with antimalarial activity**.

Zarranz et al. (2005) synthesized new series of 3-arylquinoxaline- carbonitrile derivatives (**Figure 9A**) and tested for their *in vitro* and *in vivo* activity against the erythrocytic development of *P. falciparum* with different chloroquine-resistance status. These series showed superior antimalarial activity in respect to reduced quinoxaline analogs. The best activity was observed with non-substituted QdNOs in positions 6 and 7 of the aromatic ring and with a hydrogen or chloro substituent in para position of the phenyl group (**Figure 9A**). Vicente et al. (2008) identified new compounds structurally based on 3-phenyl-quinoxaline-2-carbonitrile 1,4-di-*N*-oxide derivatives (**Figure 9B**) active against *P. falciparum*. Derivative **1** (**Figure 9B**, X = O, R7 = H) demonstrated high potency [IC(50) = 0.63 mM] and good selectivity (*SI* = 10.35), thereby becoming a new lead-compound. Marin et al. (2008) synthesized derivatives of 3-trifluoromethyl-2-arylcarbonylquinoxaline 1,4-di-*N*-oxide (**Figure 9C**) and evaluated for their capacity to inhibit the growth of chloroquine-resistant *P. falciparum* FCB1 strain in culture. Compound 7-chloro-2-(2-furylcarbonyl)-3-trifluoromethyl-1,4-quinoxaline di-*N*-oxide (**Figure 8C**, Ar = 2-Furyl, R1/R2 = Cl/H) was the most active, being almost five times more active than chloroquine. It was also 50 times more active against *P. falciparum* than toxic toward MCF7 cells. SAR showed that bioisosteric modification of phenyl group by 2-thienyl or 2-furyl subunits, R2 position must be free or occupied by a methyl group and R1 position must be free or occupied by Cl, CH_3_, OCH_3_ or CF_3_. Barea et al. (2011) synthesized new 3-amino-1,4-di-*N*-oxide quinoxaline-2-carbonitrile derivatives as acetoxybenzamides (**Figure 9D**) and sulfonamides (**Figure 9E**), and evaluated for their *in vitro* antimalarial and antileishmanial activity. Compounds with one halogenous group substituted in position 6 and 7 provide an efficient approach for further development of antimalarial and antileishmanial agents. From a series of quinoxaline analogs of chalcones in another study, compounds 1a (IC_50_= 6.2 μM; **Figure 9F**) and 2a (IC_50_= 5.8 μM; **Figure 9G**) were the most active against FCR-3 *P. falciparum* (Gil et al., 2014). SAR demonstrated the importance of an enone moiety linked to the quinoxaline ring in the search for antimalarial ligands.

The intracellular mechanism of the antiparasitic action of QdNOs is barely elucidated. Recently, Brizuela et al. (2014) shows that one QdNO-derived compound, Conoidin A [2,3-bis(bromomethyl)-1,4-dioxide-quinoxaline] (**Figure 9H**), exhibites potent lytic activity against *P. falciparum* and constitutes an irreversible Peroxiredoxin-2 (Prx2) inhibitor. Conoidin A is first shown as an inhibitor of host cell invasion by the human pathogen *T. gondii* (Carey et al., 2004), and this compound is a covalent inhibitor of *T. gondii* peroxiredoxin II (Haraldsen et al., 2009). Conoidin A can also inactivate the peroxiredoxin-1 from the human hookworm *Ancylostoma ceylanicum* by alkylating or crosslinking the catalytic cysteines, while maintaining the enzyme in the “locally unfolded” conformation (Nguyen et al., 2013). Peroxiredoxin, a thiol-dependent peroxidase, serves a critical role in converting ROS signals into a cellular response. When *Plasmodium* sp. invades red cells, it imports Prx2 from the host cell to the parasite cytosol during intraerythrocytic development in an attempt to make up for degradation of peroxides generated during cell metabolism. Therefore, treatment of erythrocytes with Conoidin A produces an unviable growth of the parasite inside, and enhances parasite sensitivity to chloroquine (Brizuela et al., 2014).

### Antitrichomonas Activity

**FIGURE 10:**
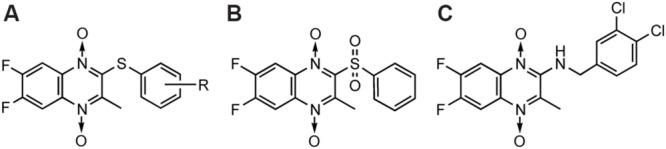
**QdNOs with antitrichomonas activity**.

Trichomoniasis is a protozoan infection of the human and bovine urogenital tracts. Metronidazole, Tinidazole and other nitroimidazoles are the most effective drugs, currently available for treatment. In the early 1980s, Glazer and Chappel (1982) reported the synthesis and the activity of a novel series of pyrido[2,3-b]quinoxaline 5-oxides against *Trichomonas foetus*. Carta et al. (2004) reported the synthesis and antitrichomonas activity of a series of 6,7-difluoro-3-methylquinoxaline 1,4-dioxides (**Figure 10A**). In particular, several 2-phenylthio derivatives resulted 20- to 30-fold more potent than the reference drug Metronidazole activity against *T. vaginalis* (SS22) *in vitro*, isolated in Italy from a case of acute vaginal trichomoniasis. For example, sulfoxide derivative (**Figure 10B**) was reported to be more effective than the reference drug metronidazole against *T. vaginalis* while one compound (**Figure 10C**) inhibited the growth of *T. vaginalis* at MIC value of 6.25 mg/mL, after 24 h of incubation.

### Antiamoebic Activity

**FIGURE 11:**
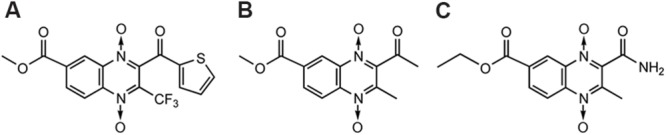
**QdNOs with antiamoebic activity**.

Amebiasis is a protozoan infection of human gastrointestinal tract caused by *Entamoeba histolytica* which results in 500 million cases and approximately 110,000 deaths annually. Out of a new series of ethyl and methyl quinoxaline-7-carboxylate 1,4-di-*N*-oxide derivatives synthesized by Duque-Montano et al. (2013), thiophene bearing motif (**Figure 11A**; IC_50_= 0.35 μM) stood out, having enhanced biological activity which was 11-fold higher than others but having selectivity index [IC_50_ (VERO cells)/IC_50_ (*E. histolytica*)] of 16.74. Compounds T-001 (**Figure 11B**) and T-016 (**Figure 11C**) showed IC_50_ values of 1.41 and 1.47 μM, respectively, with a value of selectivity index > 60.

## Antiinflammatory And Antioxidant Activities Of QdNOs

Arachidonic acid metabolism, mediated by the LOX enzyme family, leads to the generation of leukotrienes, a type of pro-inflammatory mediator involved in processes such as fever, asthma, or cardiovascular disease. Additionally, aberrant arachidonic acid metabolism is related to carcinogenesis. For example, increased LOX expression levels have been found in a wide range of cancers, including pancreatic, bladder, and breast cancer (Hofmann and Steinhilber, 2013). During the inflammation process, phagocytic leukocytes produce ROS. A number of commercially available non-steroidal antiinflammatory drugs, such as acetaminophen, salicylates, indomethacin, and nimesulide, have been shown to possess radical scavenging properties. Therefore, the development of new compounds having both antiinflammatory and antioxidant activities and being LOX inhibitors is an interesting approach for cancer prevention, treatment of chronic inflammation and other related pathological conditions.

**FIGURE 12:**
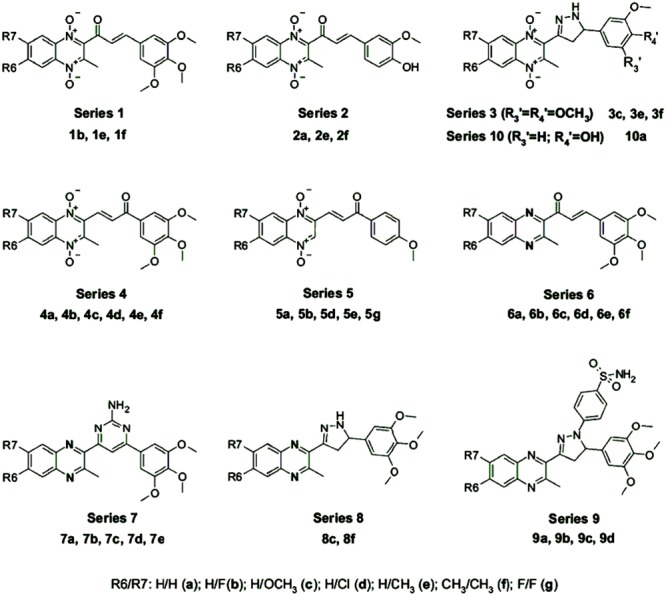
**QdNOs with antiinflammatory and antioxidant activities [adapted from Burguete et al. (2011)]**.

3-phenyl-1-(1,4-di-*N*-oxide quinoxalin-2-yl)-2-propen-1-one derivatives and their 4,5-dihydro-(1H)-pyrazole analogs were discovered to show very interesting antioxidant and antiinflammatory properties. Compound **2a** (**Figure 12**, series 2) displayed an *in vivo* antiinflammatory effect (56.1%) higher than the reference drug, indomethacin, and promising *in vitro* inhibition values of LOX (IC_50_ < 1 μM; Burguete et al., 2007). Compound **7b** (**Figure 12**, series 7) showed significant protection against carrageenan-induced paw edema, in which the *in vivo* antiinflammatory effect (41%) was similar to that of indomethacin (47%; Burguete et al., 2011).

Burguete et al. (2011) has studied the SAR which demonstrates that the radical scavenging ability needs a phenolic group (series 2) and/or a free amino pyrazoline ring (series 3, 8, and 10) in the structure (**Figure 12**). In the presence of the pyrazoline moiety, those with *N*-oxide groups in the quinoxaline ring (series 3 and 10) exhibited significantly increased reducing activity compared to their reduced analogs (series 8), resulting in increased scavenging activity. Compounds with an α, β-unsaturated ketone system (1b, 2e, 4f, 5a, 5b, 5g, and 6a–f) presented the best $O_{2}^{•–}$ scavenging activities, indicating that the olefinic moiety might play an important role in the activity by trapping the $O_{2}^{•–}$. The derivatives are also good ^∙^OH scavengers. In the inhibition of LOX, the best IC_50_ values were shown by compounds of series 7, 9, and compound 8f. Although compounds that displayed good activities of inhibition of lipid peroxidation also presented good values of inhibition of LOX, the best inhibitors of lipid peroxidation were those compounds without any substitution in the quinoxaline ring while the best inhibitors of LOX were obtained by fluoro- and methyl-substituted derivatives.

## Toxicological Effects Of QdNOs

The toxicology effects, including short- and long-term toxicities (Fang et al., 2006; Ihsan et al., 2010, 2011; Wang et al., 2010, 2011a,b, 2012), genotoxicity (Ihsan et al., 2013a,b), and photoallergic toxicity (He et al., 2006), have been extensively studied in the veterinary-used QdNOs. The liver is the main target organ of QdNOs, and OLA and MEQ show obvious toxic effect on the kidney and the adrenal gland. High dose of OLA, MEQ, CBX, and QCT can significantly inhibit the growth and development, reproductive function and embryonic development of rats, whereas CYA has minor effect. OLA, CBX, and MEQ have genotoxicity, and CBX shows obvious carcinogenic effects. QCT has certain genetic toxicity, and no genotoxicity has been detected in CYA.

Quinoxaline 1, 4-di-*N*-oxides show mutagenic and DNA-damaging effects on various organisms. Voogd et al. (1980) reported that QDX, CAX, and OLA were mutagenic on *Saccharomyces cerevisiae, Klebsiella pneumoniae, S. typhimurium* and *E. coli*, causing base-pair substitutions and frame-shift mutations. Beutin et al. (1981) found that mutagenicity of QdNOs was dependent on the presence of *N*-oxide groups, since quinoxaline, a completely reduced derivative of QDX, was not mutagenic, whereas the partially reduced quinoxaline-*N*-oxide exhibited a lower mutagenic activity than QDX. It was suggested that the mutagenicity of QdNOs resulted from the error-prone repair involved in SOS responses (Nunoshiba and Nishioka, 1989). Ihsan et al. (2013a,b) compared the genotoxic potential of QdNOs in Ames test, HGPRT gene mutation test in V79 cells, unscheduled DNA synthesis assay in human peripheral lymphocytes, chromosome aberration test, and micronucleus test in mice bone, and found that OLA, CBX, and MEQ were more genotoxic than QCT and CYA. Using alkalic comet assay, pronounced increase of DNA fragmentation were observed in Vero cells treated with CBX, OLA, and QCT (Chen et al., 2009). In contrast, DNA damage was significantly decreased after incubation with S9 mix, suggesting that the intermediate metabolites of these compounds exerted lower genotoxicity than their parent drugs. Jin et al. (2009) also found that QCT caused significant DNA fragment migration in a dose-dependent manner in human hepatoma (HepG2).

The mutagenicity of QdNOs with antiparasitic activity has also been studied. Introduction of electron-withdrawing substituents at C-6 and/or C-7 of the quinoxaline ring enhanced *in vitro* biological activity against *T. cruzi*. In addition, this led to the obtainment of non-mutagenic derivatives in both the Ames assays that were performed or their mutagenicity disappeared when performing the assay using metabolic activation (Torres et al., 2013). Using Ames test, Gabay et al. (2014) investigated the mutagenicity of an in-house chemical library of eighty five *N*-oxide containing heterocycles, and they found that in some cases, a relationship was found between the presence of *N*-oxide and mutagenicity, and in other cases, such as quinoxaline dioxides with antiparasitic activity, mutagenicity was substituent dependent.

Quinoxaline 1, 4-di-*N*-oxides can produce ROS and cause oxidative cell damage. It was found that OLA induced the increased levels of ROS and 8-OHdG in HepG2 cells, inferring that OLA exerts genotoxic effects in HepG2 cells probably through the ROS-induced oxidative DNA damage (Zou et al., 2009). Using γ-H2AX as a surrogate marker for DNA damage, Liu et al. (2012) found that MEQ treatment induced cellular DNA damage, which paralleled the chemical-induced elevation of ROS levels, and expression of the antioxidant enzyme catalase partially alleviated these MEQ-associated effects. Huang et al. (2010b) found that OLA irritated a persistent and utmost release of ROS while MEQ made a similar but weaker reaction. CYA, however, had a short and unstable release of intracellular ROS. On the other hand, quinoxalinine-2-carboxylie acid, one of the metabolites of OLA and MEQ, did not cause any significant production of ROS and showed relatively lower toxicity than its parents. Zhang et al. (2014) reported that QCT damaged the antioxidant defense abilities of HepG2 cells by reducing the activities of endogenous antioxidant enzymes, lowering glutathione concentration, and elevating malondialdehyde level. One hundred and sixty QCT-responsive genes were found to be associated with cell proliferation, glucose metabolism, oxidative stress, and apoptosis, such as NAD(P)H dehydrogenase. However, QCT metabolites (1,4-bisdesoxyquinocetone and 3-methylquinoxaline-2-carboxylic acid) showed little effects on HepG2 cells. Wang et al. (2015b) demonstrated that the rank orders of the desoxy and bidesoxy rates in rat and pig liver microsomes were QCT < CBX < MEQ < OLA < CYA and QCT < MEQ < CBX < OLA < CYA, respectively. In rats, porcine primary hepatocytes, and HepG2 cells, oxidative stress indices and DNA damage showed inverse relationships with the deoxidation rate, indicating that faster deoxidation of QdNOs results in lower DNA-damage-induced toxicity.

The molecular mechanism of cell cycle arrest and apoptosis induced by QdNOs were explored. Liu et al. (2012) report that MEQ inhibited cell proliferation by arresting cells at the G2/M phase of the cell cycle. Zou et al. (2011) demonstrated that OLA induced cell cycle arrest to the S phase and dose-dependent apoptotic cell death in HepG2 cells through a caspase-9 and -3 dependent mitochondrial pathway. Zhang et al. (2013) found that QCT induced apoptosis in HepG2 cells via activation of caspase, interaction of TNF-α and TNFR1 and modulation of the protein levels of Bid, Bax, and Bcl-2, involving the participation of p53, p38, and JNK. c-MYC-dependent activation of the mitochondrial apoptotic pathway may be associated with QCT-induced toxicity (Zhang et al., 2014). Dysregulated or excessive autophagy may lead to ‘type II programmed cell death,’ which is closely associated with apoptosis. Zhao et al. (2015) showed that OLA-induced autophagy in HepG2 cells is upregulated by Beclin 1 but downregulated by ROS-dependent c-Jun N-terminal protein kinase (JNK).

The organ toxicities of QdNOs have also been studied. Huang et al. (2009) reported the dose-dependent long-term toxicity of MEQ on adrenal gland in male rats, and its mechanism may involve in oxidative stress and steroid hormone biosynthesis pathway. Then, they found the complex interactions of MEQ metabolism, renin–angiotensin–aldosterone system, NADPH oxidase and oxidative stress in response to MEQ-induced tissue toxicity and aldosterone secretion (Huang et al., 2010a). Wang et al. (2015a) estimated the adrenal cell damage induced by QCT and its bidesoxy-QCT (B-QCT) metabolite, and suggested that its toxic effects resulted from *N*-oxide groups, and its toxic mechanism might involve the interference of the steroid hormone biosynthesis pathway. They also found ROS, the Janus kinase-signal transducer and activator of transcription (JAK/STAT) pathway, suppressors of cytokine signaling and inflammatory cytokines (TNF-α and IL-6) were involved in the liver and spleen toxicities of MEQ (Wang et al., 2011c). Ihsan et al. (2011) verified that MEQ exerted testicular toxicity by causing oxidative stress and steroidal gene expression profiles.

## Perspectives

The versatile activities of QdNOs are close related to their chemical structures. The two *N*-oxide groups in the quinoxaline ring are necessary for the antibacterial (Montoya et al., 1998; Ortega et al., 1999; Sainz et al., 1999; Zhang, 2012), antitumor (Solano et al., 2007), anti-*Trypanosoma* (Aguirre et al., 2004), and antimalaria (Zarranz et al., 2005) properties of QdNOs. Some reduced forms of QdNOs, in which the two *N*-oxide groups are absent, still possess biological activities. We assume the large side chains of these reduced quinoxaline compounds contribute more to the activities, based on the fact that in the 2-quinoxalinecarbonitrile 1,4-dioxides, the replacement of (N,N-dialkyl amino) alkyl amino chain with aromatic rigid moieties (anilines and arylpiperazines) in three position held the potency but reduced the hypoxia-selectivity (Ortega et al., 2000). The different side chains probably affect the desoxy and bidesoxy rates of QdNOs (Wang et al., 2015b), which may result in different levels of activities and toxicities.

For QdNO antitumoral compounds, when the electron-withdrawing nature of the 6(7)-substituent increases, the compound is more readily reduced and show the best activity (Monge et al., 1995b; Solano et al., 2007). This also is the case for antimycobacterial (Jaso et al., 2005), antitrypanosomal (Aguirre et al., 2004; Benitez et al., 2011), and antimalarial (Zarranz et al., 2005) activities. Interestingly, among 2-alkylcarbonyl-3- trifluoromethylquinoxaline 1,4-di-*N*-oxide derivatives, anticancer activity depends on the substituents in the carbonyl group, improving in the order: ethyl < isopropyl < tert-butyl < phenyl-ones (Zarranz et al., 2004), while among 6(7)- substituted quinoxaline-2-carboxylate 1,4-dioxide derivatives, anti-TB activity depends on the substituents in the carboxylate group, improving in the following order: tert-butyl < allyl < 2-methoxyethyl < ethyl < benzyl (Jaso et al., 2005). We speculate that the activity of alkyl group substitution must be mainly affected by the substituted carbonyl or carboxylate group, but the large benzene ring itself may contribute more to the anticancer or anti-TB activities.

In summary, SAR studies for QdNOs suggest that the position C(2) of quinoxaline should be substituted by electron-withdrawing moieties, preferably by a nitrile or an aroyl, an ester, an *N*-substituted amide group or a short brominated alkyl chain (Jampilek, 2014). The position C(3) is most often substituted by short alkyl chain (e.g., CH3, CF3) or phenyl. Substitution of C(6) or C(7) of QdNOs by Cl, F, CF_3_, or OCH_3_ is also advantageous. When the nitrile moiety is C(2) substituent, substituents in other positions should not be so much electron-withdrawing. It can be concluded that generally lipophilic and mostly electron-withdrawing substituents are preferred.

Although it has been generally accepted that QdNOs are prodrugs, transport forms of agents that are reduced to active metabolites in the body, the modes of actions of QdNOs are still indistinct. The research of the mechanism of actions of QdNOs is in progress, pushing the development of new compounds with more efficient potentials and less harmful effects. Though modes of antitumoral and antibacterial actions of QdNOs are comparatively clear, there are still some puzzles need to be explained. First, the enzyme(s), reducing and activating QdNOs to produce the free radicals, need to be identified. Since free radicals are short-lived, the location of enzyme(s) might indicate the subcellular or molecular target(s) of the drugs. Second, the type of free radical(s) and how they attack DNA or other target(s) should be identified and elucidated. Third, the network of the drug action and the interactions between drugs and organisms should be fully studied. Fortunately, the genomic and proteomic techniques have become effective ways to investigate drug action pathway and discover new drug targets. Furthermore, the research of the mode of action of QdNOs will provide clues to the study of drug resistant and toxicological mechanism, and guide more effective medication of QdNO drugs, e.g., drug combination therapy. For those modes of actions of QdNOs which are still vague, such as antiprotozoal and anticandida activity, researchers are suggested to investigate them according to the already known mechanisms and the SAR of QdNOs combined with new research tools, such as omics technology and 3D-QSAR. A deeper knowledge of the molecular targets of QdNOs is required for the development of new and more specific drugs through a rational design strategy to avoid undesirable side effects.

## Author Contributions

GC contributed to the design of the review and wrote the review. WS, CC, LG, HH, and ZL revised the review. XW and ZY contributed to the conception of the review.

## Conflict of Interest Statement

The authors declare that the research was conducted in the absence of any commercial or financial relationships that could be construed as a potential conflict of interest.

## Abbreviations

ADG: average daily gain
BTO: 1,2,4-benzotriazine 1,4-dioxide
BTZ: benzotriazinyl radical
CBX: carbadox
CYA: cyadox
DMPO: 5,5′-dimethylpyrroline 1-*N*-oxide
DSBs: double-strand breaks
*E*(1)R: one electron reduction potential
EPR: electron paramagnetic resonance
HIF: hypoxia-inducible factor
HR: homologous recombination
LOX: lipoxygenase
MDR: multidrug resistant
MEQ: mequindox
MIC: minimum inhibitory concentration
NOAEL: no observed adverse effect level
NOS: nitric oxide synthase
^∙^OH: hydroxyl radical
OLA: olaquindox
$O_{2}^{•–}$: superoxide radical
QCT: quinocetone
QdNOs: quinoxaline 1,4-di-*N*-oxides
QDX: quindoxin
ROS: reactive oxygen species
SAR: structure-activity relationship
SSBs: single-strand breaks
TB: Tuberculosis
Topo II: topoisomerase II
TPZ: tirapazamine
VEGF: vascular endothelial growth factor
2DE-MS: two-dimensional polyacrylamide gel electrophoresis combined with mass spectrometry
